# Cardiomyocyte Injury Following Acute Ischemic Stroke: Protocol for a Prospective Observational Cohort Study

**DOI:** 10.2196/24186

**Published:** 2021-02-05

**Authors:** Helena Stengl, Ramanan Ganeshan, Simon Hellwig, Edyta Blaszczyk, Jochen B Fiebach, Christian H Nolte, Axel Bauer, Jeanette Schulz-Menger, Matthias Endres, Jan F Scheitz

**Affiliations:** 1 Department of Neurology Charité - Universitätsmedizin Berlin Berlin Germany; 2 Center for Stroke Research Berlin Charité - Universitätsmedizin Berlin Berlin Germany; 3 Berlin Institute of Health Berlin Germany; 4 German Center for Cardiovascular Research (DZHK) partner site Berlin Berlin Germany; 5 Working Group on Cardiovascular Magnetic Resonance, Experimental and Clinical Research Center a Joint Cooperation Between the Charité – Universitätsmedizin Berlin, Department of Internal Medicine and Cardiology and the Max-Delbrueck Center for Molecular Medicine, and HELIOS Klinikum Berlin Buch, Department of Cardiology and Nephrology Berlin Germany; 6 German Center for Neurodegenerative Diseases (DZNE) Partner site Berlin Berlin Germany; 7 Working group on biosignal analysis, department of Cardiology Medical University of Innsbruck Innsbruck Austria; 8 Excellence Cluster NeuroCure Charité - Universitätsmedizin Berlin Berlin Germany

**Keywords:** ischemic stroke, troponin T, myocardial ischemia, myocardial injury, stroke-heart syndrome, cardiac imaging techniques, magnetic resonance imaging, Takotsubo syndrome, autonomic nervous system

## Abstract

**Background:**

Elevated cardiac troponin, which indicates cardiomyocyte injury, is common after acute ischemic stroke and is associated with poor functional outcome. Myocardial injury is part of a broad spectrum of cardiac complications that may occur after acute ischemic stroke. Previous studies have shown that in most patients, the underlying mechanism of stroke-associated myocardial injury may not be a concomitant acute coronary syndrome. Evidence from animal research and clinical and neuroimaging studies suggest that functional and structural alterations in the central autonomic network leading to stress-mediated neurocardiogenic injury may be a key underlying mechanism (ie, stroke-heart syndrome). However, the exact pathophysiological cascade remains unclear, and the diagnostic and therapeutic implications are unknown.

**Objective:**

The aim of this CORONA-IS (Cardiomyocyte injury following Acute Ischemic Stroke) study is to quantify autonomic dysfunction and to decipher downstream cardiac mechanisms leading to myocardial injury after acute ischemic stroke.

**Methods:**

In this prospective, observational, single-center cohort study, 300 patients with acute ischemic stroke, confirmed via cerebral magnetic resonance imaging (MRI) and presenting within 48 hours of symptom onset, will be recruited during in-hospital stay. On the basis of high-sensitivity cardiac troponin levels and corresponding to the fourth universal definition of myocardial infarction, 3 groups are defined (ie, no myocardial injury [no cardiac troponin elevation], chronic myocardial injury [stable elevation], and acute myocardial injury [dynamic rise/fall pattern]). Each group will include approximately 100 patients. Study patients will receive routine diagnostic care. In addition, they will receive 3 Tesla cardiovascular MRI and transthoracic echocardiography within 5 days of symptom onset to provide myocardial tissue characterization and assess cardiac function, 20-min high-resolution electrocardiogram for analysis of cardiac autonomic function, and extensive biobanking. A follow-up for cardiovascular events will be conducted 3 and 12 months after inclusion.

**Results:**

After a 4-month pilot phase, recruitment began in April 2019. We estimate a recruitment period of approximately 3 years to include 300 patients with a complete cardiovascular MRI protocol.

**Conclusions:**

Stroke-associated myocardial injury is a common and relevant complication. Our study has the potential to provide a better mechanistic understanding of heart and brain interactions in the setting of acute stroke. Thus, it is essential to develop algorithms for recognizing patients at risk and to refine diagnostic and therapeutic procedures.

**Trial Registration:**

Clinicaltrials.gov NCT03892226; https://www.clinicaltrials.gov/ct2/show/NCT03892226.

**International Registered Report Identifier (IRRID):**

DERR1-10.2196/24186

## Introduction

### Background

Elevated cardiac troponin (cTn), which is a sign of myocardial injury, frequently occurs in the early phase after an acute ischemic stroke (AIS) and is associated with poor functional outcome, especially increased mortality [1,2]. Using high-sensitivity assays, cTn is detectable in more than 90% of stroke patients; 30%-60% have at least one cTn above the assay-specific 99th percentile upper reference limit (URL) [2,3]. Approximately 5%-20% show a rise/fall pattern indicating acute myocardial injury according to the fourth universal definition of myocardial infarction (MI) [4]. In the latter group, acute coronary syndrome has to be suspected [4-6]. Following the American guidelines (American Heart Association/American Stroke Association), it is specifically recommended to measure cTn in stroke patients [7,8]. However, recommendations on how to deal with elevated cTn in the context of AIS remain vague [8]. Furthermore, expert consensus documents on practical considerations on the clinical use of cTn list ischemic stroke as one of the illnesses that leads to clinical uncertainty in the context of interpretation of elevated cTn [9].

The phenomenon of acute brain injuries (including an ischemic stroke and intracranial hemorrhage) leading to cardiac complications, including elevated cardiac enzymes, is known for a long time. In reference to AIS, it has recently been described as stroke-heart syndrome (SHS) [10-13]. However, the exact pathophysiologic background of myocardial injury (ie, cTn elevation) after stroke is not entirely understood [13,14]. Several hypotheses have been discussed. Besides concomitant acute MI caused by atherothrombotic coronary artery disease (type 1 MI), situations of oxygen supply/demand mismatch have to be considered as underlying reasons. For example, tachyarrhythmia, hypotension/shock, anemia, or respiratory failure, which are frequently seen in stroke patients, can lead to a demand ischemia [4,15]. Furthermore, systemic conditions such as sepsis or chronic kidney disease may cause or facilitate myocardial injury [16].

In the TRELAS (Troponin Elevation in Acute Ischemic Stroke) study, patients with AIS with markedly elevated cTn were significantly less likely to have a corresponding culprit lesion on coronary angiography when compared with age- and sex-matched patients with non-ST elevation acute coronary syndrome (ACS; showing no significant difference in the degree of cTn elevation) [17]. Together with the finding that half of the patients with AIS with markedly elevated cTn had no coronary artery disease at all, this implies that alternative mechanisms beyond ACS may play an important role [17]. This is supported by animal research and clinical and neuroimaging studies, suggesting that stroke-associated myocardial injury may originate from structural and/or functional interference within the central autonomic nervous system (CAN) with an overshooting sympathetic response [18-20]. On a cellular basis, it is assumed that excessive catecholamine and cortisol levels lead to an increased sarcoplasmic calcium influx with a consecutive hypercontraction of the sarcomeres, electrical instability, and metabolic and oxidative stress. Consequently, these pathological mechanisms can induce a contraction band necrosis and interstitial inflammatory reaction [12,21]. In summary, elevated troponin levels are frequent, and the underlying pathologies can be manifold, ranging from concomitant type 1 MI, demand ischemia, and chronic structural cardiac disease to systemic conditions. However, especially in situations with acute cTn elevation after AIS, the CAN seems to play an important role in the development of myocardial injury not only by triggering direct myocardial toxicity but also by facilitating situations of demand ischemia [13,14,22]. Nonetheless, the exact cascade of events remains mostly unclear, and when it comes to diagnostic procedures and treatment of affected patients, therapeutic options lack good scientific evidence [8].

### Objective

The aim of the Cardiomyocyte injury following Acute Ischemic Stroke (CORONA-IS) study is to gain mechanistic insights into stroke-associated myocardial injury. We intend to provide a detailed characterization of (1) myocardial tissue; (2) myocardial, ventricular, and atrial function; and (3) associated autonomic dysfunction by using multimodal diagnostic measures.

## Methods

### Study Design

The CORONA-IS study is an investigator-initiated, prospective, observational, single-center cohort study that aims to include 300 patients with AIS. In November 2018, the Ethics Committee of the Charité-Universitätsmedizin Berlin, Germany (EA4/123/18), approved the study. All study procedures are carried out in accordance with the principles of Good Clinical Practice and the Declaration of Helsinki. The study was pre-registered under clinicaltrials.gov, NCT03892226. All patients with AIS admitted to the hospital within 48 hours of symptom onset are listed in a hospital-based registry as part of an assessment of high-sensitivity cTn (hs-cTn) development in patients with AIS. All study patients have to fulfill the study inclusion criteria listed in Textbox 1, and all participants have to provide written informed consent (for exclusion criteria refer to Textbox 2).

**Inclusion criteria.**

- ability to provide informed consent
- age ≥18 years
- diagnosis of acute ischemic stroke and hospital admission within 48 hours of symptom onset
- visible diffusion-weighted imaging lesion in cerebral magnetic resonance imaging
- repeated measurement of high-sensitivity cardiac troponin within 24 hours of admission

**Exclusion criteria.**

- pregnancy or breastfeeding
- impaired renal function (estimated glomerular filtration rate <30 ml/min/1.73 m^2^)
- contraindications to undergo magnetic resonance imaging (eg, cardiac pacemaker, implantable cardioverter-defibrillator, and cerebral clips)
- persistent or permanent atrial fibrillation (patients with paroxysmal atrial fibrillation will be included if they are in sinus rhythm at admission or during cardiac monitoring of the stroke unit)
- ST-elevation in electrocardiogram fulfilling criteria of myocardial ischemia
- history of cardiac intervention (eg, coronary artery bypass surgery or percutaneous coronary intervention) within the last 4 weeks

Depending on serial measurements of hs-cTn during the acute hospital stay (assay characteristics: hs troponin T, Roche Elecsys, Gen 5; 99th percentile upper reference limit=14 ng/l; 10% coefficients of variation (CV) precision=13 ng/l; limit of detection=5 ng/l) and according to the fourth universal definition of MI, 3 groups are defined (Table 1) [4]. We aim to include 100 patients in each group. The cTn values are based on at least two blood exams: the first one at hospital admission and a control measurement within 24 hours after admission.

**Table 1:** Definition of the 3 groups based on patients’ high-sensitivity cardiac troponin values.

Group	Description	hs-cTn^a^ values (URL^b^=14 ng/l)
Group 1	Normal hs-cTn levels	Both hs-cTn levels ≤URL
Group 2	Chronic myocardial injury (elevated but stable cTn levels)	At least one hs-cTn level >URL but *no* rise/fall (>20%) in serial measurements
Group 3	Acute myocardial injury (dynamic elevation)	At least one hs-cTn level >URL *and* rise/fall (>20%) in serial measurements^c^

^a^hs-cTn: high-sensitivity cardiac troponin.

^b^URL: upper reference limit (=14 ng/l).

^c^Or initial cTn value ≤URL and second value >URL+increase >50% URL (ie, 7 ng/l).

**Figure 1:**
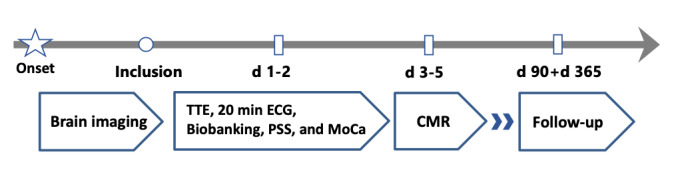
Study procedure of the Cardiomyocyte injury following Acute Ischemic Stroke study. Patients with a confirmed diagnosis of acute ischemic stroke via magnetic resonance imaging within 48 hours of symptom onset are eligible for inclusion. Patients receive a baseline visit, transthoracic echocardiography, 20-min electrocardiogram recording, blood sampling for biobanking, and cognitive testing within the first 2 days after enrolment. 3T cardiovascular magnetic resonance imaging takes place 3-5 days after symptom onset. Telephonic follow-up for cardiovascular events and functional outcomes will be conducted after 3 and 12 months. CMR: cardiovascular magnetic resonance imaging; d: day; ECG: electrocardiogram; MoCA: Motreal-Cognitive-Assessment; PSS: Perceived Stress Scale; TTE: transthoracic echocardiography; y: year.

In addition to routine clinical procedures (refer to the *Baseline Visit* section), study patients receive a baseline assessment, an additional blood draw for biobanking, 3 Tesla (3T) cardiovascular magnetic resonance imaging (CMR), transthoracic echocardiography (TTE), and a 20-min high-resolution electrocardiogram (ECG) for a comprehensive assessment of cardiac autonomic function as well as a questionnaire testing cognitive function and the perception of stress. Furthermore, 3 and 12 months after discharge, patients will be followed up via structured telephone interviews for cardiovascular events and clinical outcomes (Figure 1).

### Participants

Patients with a diagnosis of AIS, defined by a diffusion-weighted imaging lesion on magnetic resonance imaging (MRI), and hospital admission within 48 hours of symptom onset are included in the study. The study is carried out at the Department of Neurology, Charité-Universitätsmedizin Berlin, Campus Benjamin Franklin, Berlin, Germany.

### Baseline Visit

The baseline assessment of the study patients includes demographics, medical history, medication, and information about the current stroke (time of symptom onset, time of hospital admission, and treatment including thrombolysis or thrombectomy). Stroke severity is classified using the National Institutes of Health Scale Score. The degree of disability is assessed using the modified Rankin Scale score. The presence of chest pain and dyspnea at admission and before the event is documented. Cognitive function and the individual perception of stress will be assessed via 2 questionnaires: Perceived Stress Scale and Montreal-Cognitive-Assessment [23,24]. In addition, the results of routine diagnostic procedures and stroke unit monitoring (eg, vital signs, 12-lead ECG, laboratory results, cerebral computed tomography imaging/MRI, and ultrasound of the brain-supplying arteries) are recorded.

### Cardiovascular MRI Protocol

Patients receive a 3T cardiovascular MRI (CMR). The examination is performed on a 3T MR scanner (Siemens Magnetom Prisma fit 3T, Siemens) using ECG for cardiac gating.

**Figure 2:**
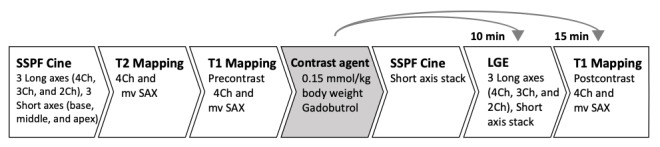
3T cardiovascular magnetic resonance imaging protocol. Workflow of the cardiovascular magnetic resonance imaging sequences conducted. 2Ch: 2-chamber view; 3Ch: 3-chamber view; 4Ch: 4-chamber view; 3T: 3 Tesla; LGE: late gadolinium enhancement; mv: midventricular; SAX: short axis view; SSPF: steady-state free-precession.

The detailed CMR protocol is depicted in Figure 2. Initially, for localizing, a half-Fourier acquisition single-shot turbo spin-echo (HASTE) sequence is conducted. Second, to evaluate the morphology and function of the left ventricle (LV) and right ventricle (RV), *steady-state free-precession cine images* (SSFP) are acquired during repeated breath-holds. Data are obtained for 3 long axes (4-chamber [4Ch], 3-chamber [3Ch], and 2-chamber [2Ch] view) and RV (imaging parameters: repetition time [TR] 45.78 ms, echo time [TE] 1.43 ms, flip angle [FA] 80°, and slice thickness 6.0 mm) and short axes stack—after contrast media application—to cover the LV (imaging parameters: TR: 44.80 ms, TE: 1.4 ms, FA: 58°, and slice thickness 7.0 mm, no gap). Furthermore, after cine long axis, 3 cine short axes (base, middle, and apex) are conducted, serving as a base for mapping imaging (imaging parameters: TR: 44.80 ms, TE: 1.4 ms, FA: 58°, and slice thickness: 7.0 mm). Motion-corrected *T2 mapping* is conducted using a fast low-angle shot (FLASH) gradient echo sequence in a 4Ch view and 3 short axis views (SAX) as basal, medial, and apical slices. T2 maps are based on images with T2 preparation times of 0/30/55 ms, slice thickness of 6.0 mm, TR of 251.49 ms, and TE of 1.32 ms.

Postcontrast imaging is performed after intravenous injection of 0.15 mmol/kg body weight Gadobutrol (Gadovist, Bayer Healthcare). *Focal fibrosis imaging* (late gadolinium enhancement [LGE]) is conducted 10 min after Gadobutrol application. LGE imaging is performed using a phase-sensitive inversion recovery sequence (PSIR) in the same slice position as cine imaging (4Ch, 3Ch, and 2Ch view; imaging parameters: TR: 750.0 ms, TE: 1.55 ms, FA: 20°, and slice thickness: 7.0 mm) as well as full coverage of the LV in a short axis package (imaging parameters: TR: 1002.4 ms, TE: 1.24 ms, FA: 55°, and slice thickness: 8.0 mm). TI was adapted to suppress the myocard.

Finally, for further myocardial tissue characterization, motion-corrected *T1 mapping* based on the Modified look-locker inversion recovery technique (MOLLI) using a 3-3-5 pattern is performed before and 15 min after contrast media application in 4Ch view and 3 short axes with basal, medial, and apical slices (imaging parameters: TR: 281.64 ms (4Ch) and 332.67 ms (SAX), TE: 1.12 ms, slice thickness: 6.0 mm, and Generalized Autocalibrating Partial Parallel Acquisition (GRAPPA) acceleration factor: 2).

Pseudonymized CMR data are transferred to the core Lab *AG Kardiale MRT* (Prof Dr J Schulz-Menger) at the Department of Cardiology, Charité Campus Buch (Berlin), for further analysis. Experienced readers (Society for Cardiovascular Magnetic Resonance level III) analyzing the MR data are blinded to the clinical data. The clinical results are provided to the study patient, and in case of pathological findings that require further diagnostics or treatment, the clinical results are provided to the patients’ treating physician.

### TTE Protocol

Patients undergo TTE on the first day after enrollment. A second TTE for evaluating dynamic changes in cardiac function is attempted on the third to fifth day thereafter. Trained physicians and trained technicians conduct the examination using the ultrasonic device *Vivid T8* (GE Healthcare). The focus of the examination is the left and right ventricular systolic as well as diastolic function and morphology. According to the guidelines of the American Society of Echocardiography, images are acquired using standard views [25]. The TTE protocol includes two-dimensional imaging, M-mode measurements, color Doppler imaging and spectral Doppler imaging (continuous-wave [CW], pulsed-wave [PW], and Doppler tissue imaging [DTI]), as well as strain imaging using a 2D-speckle-tracking technique. Systolic LV function will be defined according to the *recommendations for cardiac chamber quantification by echocardiography in adults* as normal range (left ventricular ejection fraction [LV EF] 52%-72% [male]/54%-74% [female]), mildly abnormal (42%-51% [male]/41%-53% [female]), moderately abnormal (30%-40%), and severely abnormal (<30%) [26]. Values of signs suggesting pathologic RV systolic function are defined as TAPSE (tricuspid annular plane systolic excursion) <17 mm and s’ velocity <9.5 cm/s [27]. In addition, diastolic function will be evaluated according to the criteria of the Heart Failure Association of the European Society of Cardiology [28]. An independent rater blinded to clinical information will evaluate the echocardiographic data.

### Autonomic ECG Markers

In addition to routine 12-lead ECG at admission and stroke unit monitoring, included patients receive an additional 20-min high-resolution resting ECG during the first day after enrollment using the portable medilog AR4+ device (Schiller AG). The aim is to measure specific autonomic markers periodic repolarization dynamics (PRD) and deceleration capacity (DC), reflecting sympathetic and vagal components of cardiac autonomic function in addition to standard measures of heart rate variability (HRV) in time and frequency domain [29-31]. The 7 electrodes of the high-resolution ECG are applied according to the Frank lead configuration in the 3 orthogonal axes X, Y, and Z. The examination is performed under standardized conditions (supine position, patient is not allowed to talk or change the position during the recording). For analysis, the pseudonymized ECG data are transmitted to the core lab of the academic working group *biosignal analysis* (Prof Dr A Bauer) at the cardiology department of the Medical University of Innsbruck, Austria. Members of the working group analyzing the data are blinded to all clinical information.

### Biobanking

The study protocol includes an additional blood examination for biobanking to allow future study of further potential mechanisms. Blood drawing takes place during the first day after enrollment and includes 2 EDTA (for both whole blood and plasma samples), 1 heparin, 1 coagulation sodium citrate, and 1 serum tube. Blood withdrawal, centrifugation, and processing will be conducted by a trained study nurse. Blood samples are transferred to the Central Biomaterial Bank Charité for management and storage. After processing, the stored samples consist of 5.7 mL of EDTA whole blood; 1.5 mL of citrate plasma; and 2 mL of EDTA plasma, heparinized plasma, and serum samples each. These samples will allow measurement of various potential biomarkers of interest. Dependent on further funding, we consider to determine biomarkers of cardiac injury and stress, proinflammatory markers, and markers of endothelial dysfunction (such as N-terminal B-type natriuretic peptide [NT-proBNP], midregional proatrial natriuretic peptide [MRproANP], Copeptin, interleukin-6, interleukin-1β, Soluble suppression of tumorigenicity 2 [sST2], and monocyte chemoattractant protein-1 [MCP-1]). Furthermore, we consider exploring whether patients with signs of stroke-associated myocardial injury present distinct miRNA-pattern. Finally, the design allows future cooperation with other research groups.

### Follow-Up Telephone Interview

A follow-up regarding major cardiovascular events takes place 3 and 12 months after enrollment via telephone interview and is conducted by a trained participant of the research group. A major cardiovascular event is defined as the occurrence of transient ischemic attack and ischemic stroke, intracranial hemorrhage, MI, coronary artery bypass surgery or percutaneous coronary intervention, new atrial fibrillation, hospitalization for heart failure, and death. The functional outcome is assessed using the modified Rankin Scale. In case of death, the date of death is recorded using information from registration offices. In the case of cardiovascular events, medical records will be requested from the treating physician/institution. Furthermore, Charité records will be screened for readmission or further treatment. In case of unclear loss to follow-up, mortality status will be retrieved from the residents’ registration office.

### Study Outcomes

**Figure 3:**
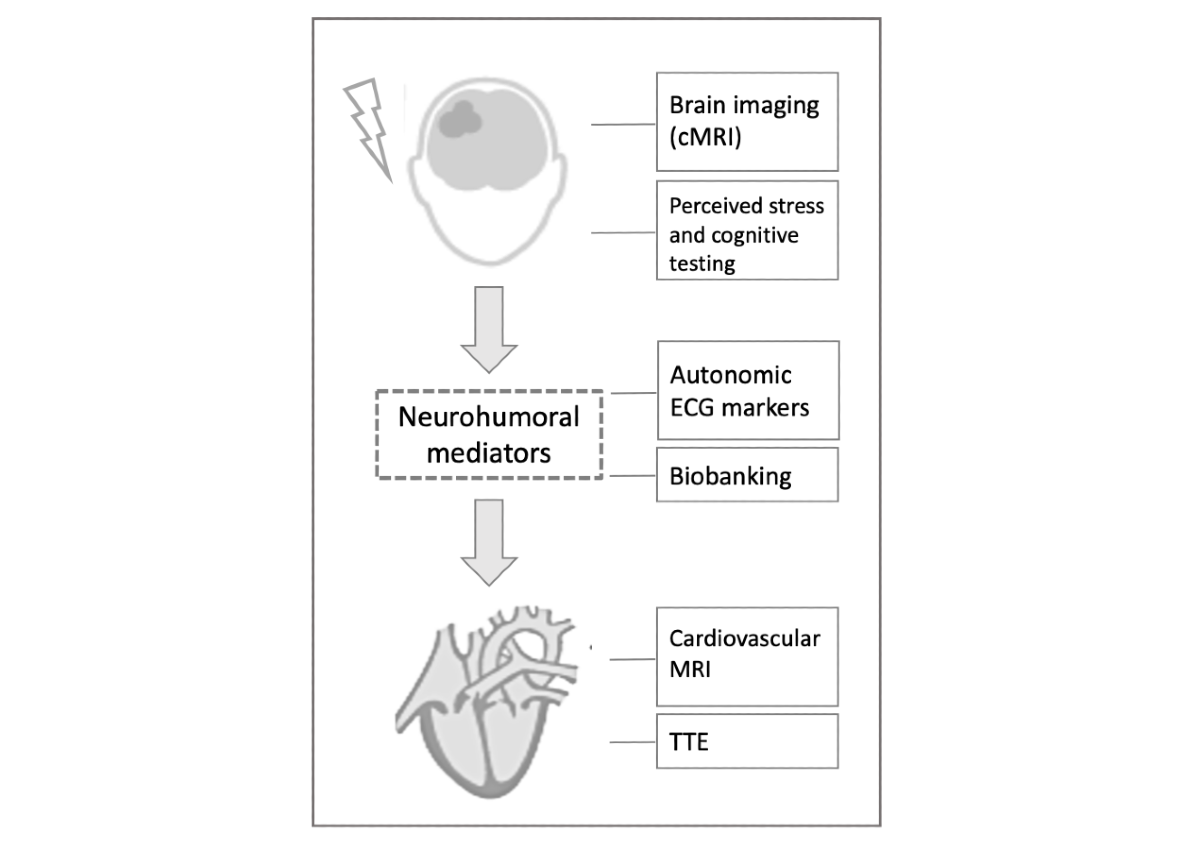
Diagnostic assessment of the Cardiomyocyte injury following Acute Ischemic Stroke study. Illustration of the target points of the multimodal diagnostic workup to provide a thorough phenotyping of patients with stroke-associated myocardial injury. cMRI: cerebral magnetic resonance imaging; ECG: electrocardiogram; MRI: magnetic resonance imaging; TTE: transthoracic echocardiography.

Our main hypothesis is that the development of stroke-associated myocardial injury in patients with AIS is based on a stroke-related interference in the CAN resulting in myocardial tissue alterations and dysfunction (ie, stroke-induced "myocardial stunning") [13]. Using a systemic and multimodal diagnostic approach, we aim to provide a detailed characterization of myocardial tissue, cardiac function, and autonomic cardiac regulation (Figure 3). Thus, outcome measures are primarily based on cardiac tissue characterization via CMR, functional assessment using TTE and CMR measurements, and values of specific autonomic ECG markers. Textbox 3 shows the detailed outcome measures. In summary, as we assume that patients with stroke-associated myocardial injury show a Takotsubo syndrome (TTS)/stress cardiomyopathy pattern of lesions in the myocardium, we will focus on the presence of wall motion abnormalities together with myocardial edema (T2 mapping) but without corresponding LGE in CMR [32,33]. Left ventricular dysfunction and wall motion abnormalities will be measured via cine imaging in CMR and TTE. As a correlate of chronic myocardial injury, we further assess myocardial fibrosis/scar via LGE, diffuse fibrosis via T1 mapping, and extracellular volume fraction (ECV%) [34]. To facilitate the differentiation between stroke-induced and coronary-mediated myocardial injury, we evaluate typical CMR signs suggesting a recent MI (ie, presence of co-occurring LGE and acute edema in CMR). Infarcted myocardium will be defined as a region with a mean signal intensity >5SDs relative to the remote uninjured myocardium on LGE images [35].

**Study endpoints.**

Primary outcome measures

- Frequency of Takotsubo syndrome pattern on cardiovascular magnetic resonance imaging (CMR); consisting of wall motion abnormalities together with myocardial edema (T2 mapping) but without late gadolinium enhancement
- Frequency and extent of myocardial edema
- Frequency of recent myocardial infarction on CMR
- Frequency and extent of ischemic and nonischemic myocardial fibrosis according to late gadolinium enhancement imaging and according to extracellular volume fraction on T1 mapping
- Frequency of left ventricular dysfunction in CMR (ie, ejection fraction and end diastolic left ventricular volume)
- Frequency of impaired left ventricular systolic and diastolic function in the transthoracic echocardiography

Secondary outcome measures

- Frequency of pathologic values of Periodic Repolarization Dynamics (PRDs) and Deceleration Capacity (DC; PRD≥5.75 deg²; DC≤2.5 ms)
- Frequency of values corresponding to *high perceived stress* in the Perceived Stress Scale (values ranging from 27 to 40)
- Frequency of cardiovascular events after 3 and 12 months
- Functional outcome after 3 and 12 months assessed using the modified Rankin Scale.

### Sample Size Calculation and Statistical Analysis

Regarding the primary hypothesis and based on the published literature, we expect a rate of acute myocardial edema on T2 mapping in CMR in approximately 15% of patients with acute myocardial injury [17,36]. In the comparison groups with no or chronic myocardial injury, we expect a significantly lower rate (approximately 2%) presenting with acute myocardial edema [37].

To show a significant difference between the groups (two-sided α=.05), a sample size of 48 patients per group is required to reach a power of 80% and 89 patients per group for a power of 90%. Taking into consideration that in previous studies, due to impaired compliance or technical problems, the complete protocol of CMR could be realized only in approximately 85% of the study patients, we aim to include approximately 100-105 patients in each group.

Group comparisons (when comparing between the 3 groups) of the primary and secondary outcome measures (frequencies of specific alterations in CMR, TTE, and ECG) will be conducted using the chi-square test for categorical variables and, in case of continuous variables, using one-way analysis of variance or Kruskal-Wallis test, as appropriate. When comparing 2 groups (group 3 vs group 1 or group 3 vs group 2), Student *t* test will be used to compare continuous data. Logistic regression analyses will be used to calculate odds ratios and 95% CI to examine the association between elevated hs-cTn levels and the presence of specific structural and functional cardiac alterations in CMR and TTE. Multiple regression analyses using backward selection will be used to identify factors associated with certain myocardial alterations or ECG findings.

## Results

Screening started in January 2019. After the initial pilot phase, the first patient was enrolled in April 2019. We estimate a recruitment period of approximately 3 years to enroll 300 patients with the complete CMR protocol. At the time of submission, 107 patients had been included. The final results are expected in 2023.

## Discussion

### Overview

This prospective, observational CORONA-IS study aims to clarify the underlying pathobiology of stroke-associated myocardial injury. The observation that patients with acute, severe neurological events often develop cardiac complications is well known and has been described as SHS or brain-heart syndrome [13]. Although there are strong indicators, suggesting that a stroke-induced dysregulation of the CAN leads to functional and structural cardiac alterations, many aspects of the pathophysiology remain unknown, and so far, no diagnostic or therapeutic algorithms for the treatment of these patients are available. Therefore, the aim of the CORONA-IS study is to explore and clarify the pathway from the brain to the heart, focusing on the crucial role of the autonomic nervous system and the cardiac phenotype.

The first goal is to visualize downstream cardiac mechanisms using CMR and TTE. We expect stroke patients with acute myocardial injury to show a higher rate and a different pattern of myocardial edema than patients with normal cTn. More precisely, we expect a myocardial edema (in T2 mapping in CMR) with wall motion abnormalities but without LGE [36,38]. This combination of edema without LGE is also seen in TTS, a condition that is in turn associated with an increased sympathetic stimulation [39]. TTS typically occurs following an emotionally or physically triggering event, but it can also develop after an acute neurologic illness [40]. In addition, we aim to assess alterations suggesting an acute or recent MI in the different groups. So far, several studies have applied CMR in stroke patients but mostly as part of a diagnostic workup to determine possible cardioembolic etiology in cryptogenic stroke [41-43]. For example, the HEBRAS (HEart and BRain interfaces in Acute ischemic Stroke) study will determine whether an enhanced diagnostic MRI workup (including CMR) combined with prolonged Holter monitoring will increase the detection rate of pathologic cardiac findings in patients with AIS [44]. To date, myocardial tissue characterization in patients with stroke-associated myocardial injury has not been investigated via CMR.

Besides structural alterations of the myocardium, we further aim to clarify whether patients with AIS and stroke-associated myocardial injury show—especially transient—functional cardiac alterations. Cardiac dysfunctions, including wall motion abnormalities or reduced EF, are often seen in patients with ischemic stroke and other acute severe neurologic conditions [1,45,46]. Regarding our study population, we expect to see higher rates of changes in left ventricular systolic and diastolic functions in patients with dynamic troponin elevation.

The second aim of the study is to investigate the role of CAN in the development of stroke-associated myocardial injury. There are different ways to display the influence of CAN on the cardiovascular system. Invasive diagnostic methods with direct recording of neural activity are not feasible in clinical settings. Noninvasive methods include for instance measurement of baroreceptor sensitivity or HRV. Reduced HRV and impaired baroreceptor sensitivity are associated with higher stroke severity and worse clinical outcomes [47,48]. However, these diagnostic tools represent only the combined sympathetic and parasympathetic influence on the cardiovascular system. There is evidence that increased sympathetic nervous activity can lead to destabilization of the myocardial repolarization phase [49]. In the CORONA-IS study, we will use the novel ECG markers, PRD, and DC*.* PRD assesses rhythmic modulations of cardiac repolarization in the low-frequency spectral range (≤0.1 Hz) [31,50]. Experimental and clinical evidence suggests that these low-frequency alterations are caused by phasic efferent sympathetic activity. DC is an integral measure of deceleration-related oscillations of the heart rate and primarily reflects parasympathetic activity [51]. PRD alone and in combination with DC have been shown to be a strong and independent predictor of sudden cardiac death in patients with MI [30,31,52]. To date, these markers have not been investigated in patients with AIS. They could provide important information regarding the assumed dysfunction of the CAN causing stroke-associated myocardial injury. It has to be kept in mind that these noninvasive measures can only serve to display an association between altered autonomic cardiac control and the presence of myocardial injury in stroke patients. To show a direct causation, further studies with nonobservational designs will be necessary. As the clinical differentiation between concomitant MI and stroke-associated myocardial injury is difficult, we aim to investigate whether specific biomarkers can help distinguish between both conditions. Therefore, we conduct thorough biobanking to evaluate the role of various potential biomarkers.

### Limitations

Some limitations of the study will need to be considered. First, as the aim of CORONA-IS is to investigate patients with stroke-associated myocardial injury (ie, SHS), it is necessary to avoid including patients with clearly coronary-mediated myocardial ischemia. Hence, patients with signs of a concomitant or recent MI (ie, typical alterations in the ECG, such as ST elevations or a new left bundle branch block, as well as a recent coronary artery bypass surgery or percutaneous coronary intervention) will be excluded. Second, as CMR and the assessment of autonomic ECG markers depend on a rhythmic heartbeat, patients with persistent or permanent atrial fibrillation will not be included, even though they may be prone to develop stroke-associated myocardial injury. Third, specific contraindications to undergo CMR (eg, certain metallic implants, claustrophobia, or physiologic constitution such as severe obesity or an inability to stay in the supine position) may lead to an underrepresentation of these patients in the study. To correct for potential selection bias in the final analysis, the screen log of the study will be analyzed at the end of data collection to assess whether the rate of excluded patients due to CMR contraindications differed among the 3 groups. Finally, considering the necessity of giving informed consent to participate in the CORONA-IS trial, patients with large infarctions and aphasia may also be underrepresented.

### Conclusions

In summary, the CORONA-IS study aims to provide a deep phenotyping of patients with stroke-associated myocardial injury by using different diagnostic tools, such as 3T CMR, TTE, specific novel autonomic ECG markers, and different blood biomarkers. The goal of this prospective, observational study is to develop a better understanding of the characteristics and the pathophysiology of stroke-associated acute myocardial injury (SHS) to identify patients at risk and improve diagnostic and therapeutic procedures.

## Abbreviations

2Ch: 2 chamber
3T: 3 Tesla
3Ch: 3 chamber
4Ch: 4 chamber
ACS: acute coronary syndrome
AIS: acute ischemic stroke
CAN: central autonomic nervous system
CMR: cardiovascular magnetic resonance imaging
CORONA-IS: Cardiomyocyte injury following Acute Ischemic Stroke
cTn: cardiac troponin
DC: deceleration capacity
ECG: electrocardiogram
EF: ejection fraction
FA: flip angle
HRV: heart rate variability
hs-cTn: high-sensitivity cTn
LGE: late Gadolinium enhancement
LV: left ventricle
MI: myocardial infarction
MRI: magnetic resonance imaging
PRD: periodic repolarization dynamics
RV: right ventricle
SAX: short axis view
SHS: stroke-heart syndrome
TTE: transthoracic echocardiography
TTS: Takotsubo syndrome
